# Prevalence and predictors of arthritis among adults in a rural set-up in Kenya: a cross-sectional study

**DOI:** 10.11604/pamj.2024.47.158.42890

**Published:** 2024-04-03

**Authors:** Shem Nyarunda Kinara, Harun Mbugua Kimani, Gordon Oluoch Ogweno

**Affiliations:** 1 Department of Family Medicine Community Health and Epidemiology, Kenyatta University, Nairobi, Kenya,; 2 Department of Medical Physiology, Kenyatta University, Nairobi, Kenya

**Keywords:** Prevalence, predictors, risk factors, arthritis, community-based study, Kenya

## Abstract

**Introduction:**

arthritis is a significant public health problem affecting many people globally. Exposure to various risk factors puts individuals at risk of developing arthritis. Therefore, this study aimed to assess the prevalence and predictors of arthritis among residents of a rural set-up in Nyamira County, Kenya.

**Methods:**

a community-based cross-sectional study design was employed. Simple random sampling was utilized to select households from a household list. All the residents of the sampled household above 40 years were included. Descriptive analysis was done to describe the study population. Bivariate and multivariate analysis was also done to identify statistically significant arthritis-related variables.

**Results:**

the prevalence of arthritis was 44.6%. Previous joint injury/infection [AOR=2.74; 95%CI=1.59-4.77; p<0.001], being unemployed [AOR=2.77; 95%CI=1.50-5.21; p=0.001], age above 51 years, and hypertension [AOR=1.90; 95%CI=1.03-3.53, p=0.040] were associated with an increased risk of arthritis. Conversely, being male [AOR=0.42; 95% CI=0.22-0.75; p=0.005], standing for > 2 hours [AOR=0.48; 95%CI=0.29-0.81; p=0.006], and constant shifting from sit to stand positions [AOR=0.45; 95% CI=0.26-0.76; p=0.003] were associated with a lower risk of arthritis. Most participants (75%) had an arthritis knowledge score of more than 66%.

**Conclusion:**

the study found a high prevalence of arthritis in the community. Arthritis was strongly associated with various risk factors under study. Therefore, there is a need to take preventive measures for modifiable factors to enhance a reduced prevalence of arthritis.

## Introduction

Osteoarthritis (OA) and rheumatoid arthritis (RA) are a significant public health problem affecting many people globally [1,2]. These conditions are often associated with pain, stiffness, reduced range of motion, and joint deformities [3]. Globally, approximately 7.6% had osteoarthritis in 2019, an increment of 132.2% of the total cases since 1990 [1]. The trend is also replicated in rheumatoid arthritis, where approximately 20 million prevalent cases and 1.2 million incident cases affected people globally as of 2017 [2]. An epidemiological analysis of RA incidence from 1990 to 2019 found an increment of 6.47% [4]. In Africa, the burden of OA ranges from 0.4% to 82.7% [5,6], while that of RA varies from 0.1% to 2.5% [5]. A Community Oriented Programme for the Control of Rheumatic Diseases in Africa in two sub-Saharan Africa (SSA) countries found the prevalence of osteoarthritis to be 6.1% and 22.1% in peri- and semi-urban Nigeria, respectively [7,8] and 36.8% in rural Congo [9]. Conversely, the exact prevalence of RA in SSA is unknown; however, it is statistically projected to be 135.7 to 231.1 per 100,000 people [2]. In Kenya, there is limited data on the spectrum of rheumatic diseases. Despite that, a hospital study found the prevalence of OA to be 9.6% [10], while a population-based survey conducted in urban slums of Nairobi indicated that approximately 42.6% had arthritis symptoms, with 25.3% having an arthritis diagnosis [11]. Together, these findings suggest an increasing burden of arthritis within our populations.

The rise in the incidence of RA patients may be associated with increased survival [12], early detection, advanced medical practices, environmental exposures, and genetic factors [13]. Africa's rheumatic diseases are probably higher than other parts of the globe in terms of morbidity and mortality, as patients often present at a later stage of disease [14,15]. With an ageing global population and an increase in arthritis attributed to activity limitation [16], the economic and health burden will increase significantly [17]. This may lead to a loss of dependence, lack of social participation, workplace absenteeism, and reduced psychological well-being [17,18]. Similarly, arthritis risk factors such as occupational exposures [19] and multimorbidity of non-communicable diseases [20] are on the rise in low- and middle-income countries (LMIC), and Kenya is one of them. This can lead to a high prevalence of arthritis cases. According to the Nyamira county integrated development plan 2018 -2019, arthritis was the second cause of morbidity [21]. Therefore, to comprehend predisposing arthritis risk factors among people living in LMIC, there is a need for population-based studies and evidence-based prevention strategies.

This study aimed to assess the prevalence and predictors of arthritis among residents of a rural set-up in Nyamira County, Kenya, where arthritis was the second.

## Methods

**Study design:** we conducted a cross-sectional study using a household survey among residents living in rural Nyamira County. This study was conducted between the 1^st^ and 30^th^ of January 2023 by investigators affiliated with Kenyatta University.

**Study setting:** the Tombe area has 2,270 households and 8,912 people in Nyamira County, Kenya. Nyamira County has a population size of 605,576 [22].

**Sample size determination:** this study utilized Fisher's unlimited and finite population formula.

Unlimited population:

Finite population:

Where Z is the z score set at 1.96, which corresponds to a 95% confidence level, εis the margin of error set at 5%, Nis household population size for the Tombe location (2270) [22], and x̂ is the population proportion of the primary outcome (arthritis) set at 21.5% [23]. After finite population correction and adjustments for spoilt questionnaires (10%), the total sample size yielded 258 households.

**Participants:** all participants above forty years in the randomly sampled households were selected to participate in the arthritis household survey from the thirty-three selected community units. Trained research assistants conducted interviews with 307 persons who consented to participate.

### Variables/measures

**Arthritis status: doctor-diagnosed and symptom-based criterion:** arthritis status, the outcome variable, was measured using a doctor's diagnosis and an arthritis symptom-based criterion. Doctor-diagnosed arthritis was based on participant responses to the question, *"Have you ever been diagnosed with/told by a healthcare professional you have arthritis (a disease of the joints or, by other names, rheumatism or osteoarthritis)?"* Those who responded “No” were further screened with an arthritis symptom-based algorithm developed by the WHO SAGE study team [24] to determine the pattern of symptoms indicative of osteoarthritis instead of symptoms most likely to show inflammatory arthritis as shown in Table 1.

**Table 1:** symptom-based questions and related algorithms to ascertain prevalent arthritis

Question number	Question text and algorithm
1	During the last 12 months, have you experienced pain, aching, stiffness or swelling in or around the joints (like arms, hands, legs or feet) that were not related to an injury and lasted for more than a month?
2	During the last 12 months, have you experienced stiffness in the joint in the morning after getting up from bed or after a long rest of the joint without movement?
3	Did this stiffness last for more than 30 minutes?
4	Did this stiffness go away after exercise or movement in the joint?
*Algorithm*	*If a participant responded with 'yes' to questions 1 and/or 2 and responded with 'no' to questions 3 and 4, then the participant was categorized as most likely having osteoarthritis (rather than inflammatory arthritis)*

**Correlates of arthritis:** this study's independent variables were sociodemographic, occupation-related stressors, lifestyle, and medical risk factors. The sociodemographic factors assessed included age (40-50, 51-60, 61-70, 71+), gender (male or female), education (high school and above, below primary school, or no formal schooling), marital status (married/cohabiting or single/divorced/ widowed/separated), social health insurance (No or Yes), employment status (self-employed/ employed or unemployed), household size (1-4 or 5+), and area of residence (rural or town centre). The medical risk factors assessed included previous joint injury (Yes or No) and comorbidities (Yes-specify or No). On the other hand, the occupational-related stressors explored entailed repetitive joint movements (standing for >2 hours or constant shifting from sit to stand position), carrying heavy loads ≥10kg, walking for >3km, and tea picking being measured as (Daily/regularly or Never/Rarely). The lifestyle risk factors assessed involved exercise (Yes or No). Finally, the level of knowledge on arthritis was assessed using a validated knowledge tool adopted from a study on knowledge of symptomatic knee osteoarthritis among railway workers [25]. The tool had thirty-five components assessing knowledge of risk factors, signs and symptoms, disability, preventive measures, and aspects to relieve arthritis symptoms.

**Statistical methods:** data was analyzed using the R version 4.2.2. Univariate analysis was done, where data was analyzed into frequency and percentages. Bivariate analysis was conducted to estimate the association between dependent (arthritis) and independent variables (sociodemographic, medical, occupational, and lifestyle factors) in a statistically significant manner (p≤0.05). Under the bivariate settings, the association between categorical variables was assessed using Pearson's Chi-Square test. The association between categorical variables such as gender, the highest level of education, and employment status, among others and continuous variables, e.g., age and household size, were done using two-sample t-tests, assuming Gaussian distribution for continuous variables. A two-sample Wilcoxon rank sum test was used for the association between categorical and continuous variables, which are not normally distributed. All two-way variable interactions for significant risk factors with arthritis were investigated through multivariate logistic regression models. We also performed a survival analysis to assess the median age of developing arthritis among females and males.

**Ethical approval and consent to participate:** ethical approval to carry out this research was obtained from the Kenyatta University Ethical Review Committee (KUERC). Besides that, informed consent was sought from all individuals who agreed to participate in the study. This is after the procedures, benefits, disadvantages, and objectives of this study had been explained to them.

## Results

**Respondent´s characteristics:** the respondent characteristics of the 307 participants included are shown in Table 2. Most participants were aged 40-50, 112(36.5%), and many had a household size of between 1 and 4 people, 172 (56.0%). Regarding other variables, 197 (64.2%) were females, 135 (44.0%) had the highest level of education, 239 (77.9%) were married/cohabiting and 168 (54.7%) had a social health insurance. Most people exercised 197 (64.2%) and lived in rural set-ups, 245 (79.8%), as opposed to town centres within the community.

**Table 2:** respondent characteristics

Characteristic	Total (N=307)	Percentage %
**Age group**		
40-50	112	**36.5**
51-60	64	20.8
61-70	75	24.4
71 +	56	18.2
**Gender**		
Female	197	**64.2**
Male	110	35.8
**Education**		
High school and above	135	**44.0**
Below primary school	113	36.8
No formal schooling	59	19.2
**Marital status**		
Married/Cohabiting	239	**77.9**
Single/Divorced/Widowed/Separated	68	22.1
**Social health insurance**		
No	139	45.3
Yes	168	**54.7**
**Current employment status**		
Self-employed/Employed	232	**75.6**
Unemployed	75	24.4
**Household size**		
1-4	172	56.0
5+	135	44.0
**Area**		
Rural	245	79.8
Town center	62	20.2
**Exercise**		
No	110	35.8
Yes	197	64.2

**Prevalence of arthritis within the community:** the proportion of respondents with arthritis among the 307 participants recruited in the study was 137/307. This gave a prevalence of 44.6%.

**Figure 1:**
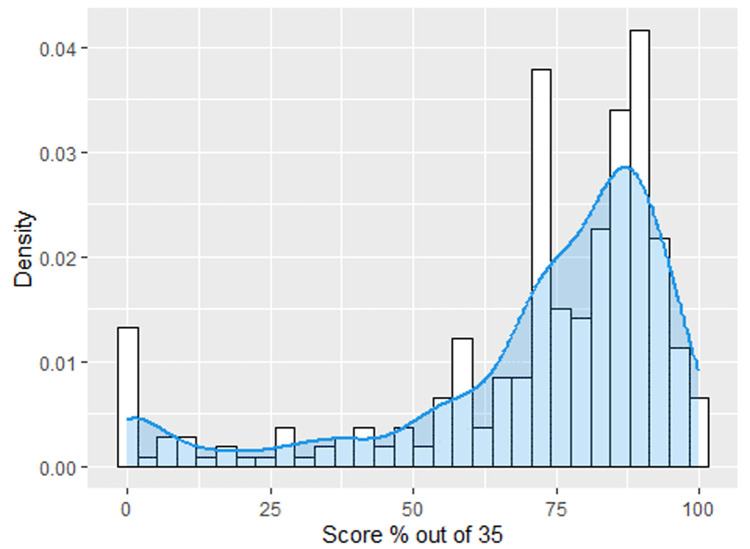
distribution of knowledge of arthritis

**Knowledge on arthritis:** knowledge of arthritis was assessed based on thirty-five items. From the density plot (Figure 1), 75% of the participants scored more than 66%, making the arthritis knowledge score right-skewed.

**Predictors of arthritis - a multivariate logistics regression analysis**: Table 3 shows the predictors of arthritis after a multivariate logistics regression analysis. Age above 51 plus years, unemployed (AOR 2.77 [1.50-5.21], p=0.001), staying in a town centre (AOR 2.02 [1.08-3.83], p=0.028), previous joint injury/infection (AOR 2.74 [1.59-4.77], p=0.001) and having hypertension (AOR 1.90 [1.03-3.53], p=0.040) had increased odds of developing arthritis. On the other hand, being male (AOR 0.42 [0.23-0.75], p=0.005), standing for >2 hours daily (AOR 0.48 [0.29-0.81], p=0.006) and constantly shifting between sit to stand positions (AOR 0.45 [0.26-0.76], p=0.003) were found to have a lower risk of arthritis.

**Table 3:** predictors of arthritis - a multivariate logistics regression analysis

Variable/category	Unadjusted ORs (95% CI)	P-value	Adjusted OR (95% CI)	P-value
**Age**				
40-50	-		-	
51-60	2.07 (1.09-3.95)	p=0.026	**2.10 (1.06-4.20)**	**p=0.034**
61-70	2.86 (1.56-5.31)	p=0.001	**2.45 (1.20-5.07)**	**p=0.015**
71 +	4.50 (2.30-9.05)	p<0.001	**4.67 (1.93-11.72)**	**p=0.001**
**Gender**				
Female	-		-	
Male	0.59 (0.36-0.95)	p=0.030	**0.42 (0.23-0.75)**	**p=0.005**
**Education**				
High school and above	-		-	
Below primary school	1.60 (0.96-2.68)	p=0.071	1.54 (0.86-2.80)	p=0.149
No formal schooling	2.84 (1.52-5.39)	p=0.001	1.40 (0.65-3.02)	p=0.393
**Social health insurance**				
No	-		-	
Yes	1.62 (1.03-2.57)	p=0.038	1.62 (0.96-2.78)	p=0.074
**Employment status**				
Self-employed/Employed	-		-	
Unemployed	3.91 (2.26-6.94)	p<0.001	2**.77 (1.50-5.21)**	**p=0.001**
**Area**				
Rural	-		-	
Town center	1.67 (0.96-2.95)	p=0.072	**2.02 (1.08-3.83)**	**p=0.028**
**Previous joint injury**				
No	-		-	
Yes	2.76 (1.70-4.51)	p<0.001	**2.74 (1.59-4.77)**	**p=0.001**
**Hypertension**				
No	-		-	
Yes	2.48 (1.45-4.29)	p=0.001	**1.90 (1.03-3.53)**	**p=0.040**
**Standing for >2 hours/day**				
No	-		-	
Yes	0.46 (0.29-0.74)	p=0.001	**0.48 (0.29-0.81)**	**p=0.006**
**Constant shifting: sit-stand daily**				
No	-		-	
Yes	0.45 (0.28-0.72)	p=0.001	**0.45 (0.26-0.76)**	**p=0.003**
**Carrying heavy loads >10kg**				
Daily/regularly	-		-	
Never/Rarely	1.72 (1.08-2.75)	p=0.023	1.12 (0.60-2.07)	p=0.721
**Walking for > 3km**				
Daily/Regularly	-		-	
Never/Rarely	2.04 (1.27-3.30)	p=0.004	1.54 (0.86-2.76)	p=0.146
**Tea picking**				
Daily/Regularly	-		-	
Never/Rarely	1.87 (1.19-2.97)	p=0.007	1.56 (0.89-2.71)	p=0.117

**Figure 2:**
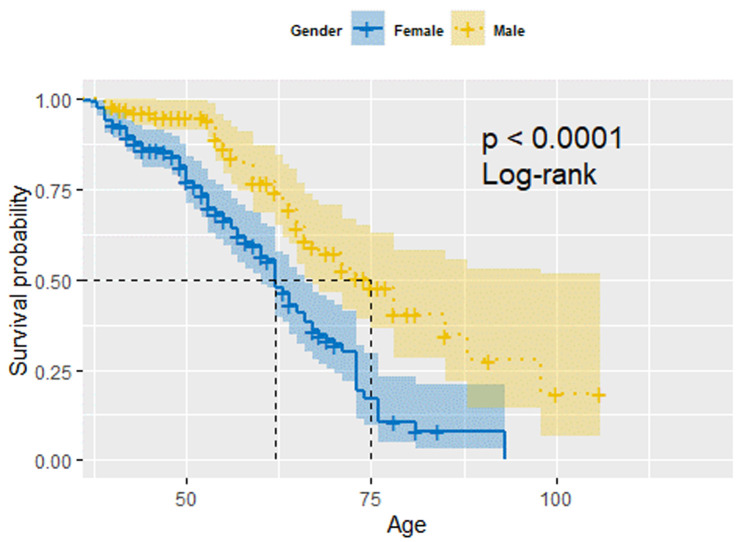
survival analysis of arthritis based on gender and age

**Survival analysis of arthritis based on gender and age:** Figure 2 shows the survival probability of arthritis based on gender the probability of developing arthritis increases as age advances. Our survival curve starts at age 40 since that was the minimum age in our data set. There is a significant difference between males and females, with log-rank test p-value <0.001, where females are more prone to developing arthritis than males. Moreover, females have a short median age of 63 years compared to 75 years in males.

## Discussion

This study determined the prevalence and predictors of arthritis among adults living in a rural set-up, Kenya. We found out that there was a high prevalence of arthritis. Additionally, age, being male, the unemployed, staying in a town center, previous joint injury, having hypertension, standing for >2hours daily, and constant shift from sit to stand positions to be significant predictors of arthritis.

The current study revealed that 137 (44.6%) had arthritis. The prevalence of the current study was higher than a Kenyan urban slums study (25.3%) [11] and a WHO study in six low and middle-income countries (China, Ghana, Russia, India, Mexico, and South Africa) (21.5%) [23]. The difference in prevalence with the urban slums study may be related to the composition of the study participants, methodology, and the research targeting participants above 60 years. On the other hand, variations with the WHO study may be related to differences in working environments, geographical locations, and diverse populations. However, our prevalence was within the range of a systematic review and meta-analysis of arthritis in Africa [5]. The high prevalence within our communities implies the need of evidence-based prevention strategies. Besides that, this study showed a high level of arthritis knowledge score. This score exceeded a survey of Malaysian railway workers (53.6%) [25]. The difference in the findings may be related to the continued effort by the county Department of Health to create awareness of arthritis, being the second cause morbidity as of 2017 [21]. Knowledge empowerment of patients with chronic diseases allows for purposeful use of the knowledge about the condition to achieve specific health related goals [26].

From this study, it was found that the age of the study participant was a significant predictor of arthritis. People above 71 years had the highest odds of developing arthritis compared to those between 40 and 50. These findings are similar to studies in six low and middle-income countries [23], the US National Health Interview Survey [27], and an OA systematic review and meta-analysis [28]. When comparing females and males, our survival analysis showed that females had a shorter median age of developing arthritis than males. Subsequently, being male was associated with lower odds of developing arthritis, corresponding to established consensus [28,29]. However, there is need for a longitudinal study to perform the arthritis survival analysis. Our study being a cross-sectional study might have faced recall bias. Besides that, being unemployed was associated with a high risk of arthritis. This concurs with a risk factors study that found early retirement, unemployment, unskilled labour, manual work, and lower-than-average income are linked with the development of osteoarthritis [30]. Moreover, unemployed people tend to do heavy physical activities, which increases the risk of developing musculoskeletal disorders [31]. To avoid increased incidences of osteoarthritis in low and middle income countries, there is need for review and prevention of occupational exposures to ergonomic risk factors [32].

The majority of the participants with arthritis were from remote parts rather than town centres. Our findings concur with a Chinese population study that found a high prevalence of arthritis among those living in rural areas [28]. Similarly, a United States population study found that rural residents had arthritis as a highly prevalent health condition [16]. Rural areas are highly prevalent because of recognized risk factors, including older age, obesity, and lower socioeconomic status [16]. Unlike the two previous studies, obesity is not a problem in the remote areas of Kenya; it affects people living in urban centres [33]. Of importance is that many of the participants (64.2%) in the study exercised. Exercise in combination with other arthritis therapies can help improve physical activity as well as reduce joint pain [34]. A previous joint injury or infection was recognized as a significant predictor of arthritis. This is consistent with previous research studies that have shown joint injuries can significantly enhance the risk of osteoarthritis, depending on the patient's age and time from the onset of injury [28,35,36]. Continuous release of inflammatory mediators into the joint may lead to the arthritis pathogenesis [37,38].

Hypertension was found to have a strong association with arthritis. Comparing other studies, a US National Health and Nutrition Examination Survey (1999-2018) found that individuals with hypertension were indeed at a higher risk of developing arthritis compared to those without hypertension. It also showed that hypertensive individuals had a 27% greater likelihood of developing arthritis during the study period [39]. An intricate interplay exists between arthritis and comorbidities, including cardiovascular diseases, diabetes, and asthma [4]. These findings underscore the importance of considering hypertension as a potential risk factor for arthritis and highlight the need for further research into the mechanisms underlying this association.

Standing for >2 hours per day was found to be protective. This finding is against a systematic review and meta-analysis study on risk factors of arthritis that showed standing for >2 hours daily was associated with the development of arthritis [28]. The difference in findings may be related to the heterogeneous characteristics of our population as opposed to the homogeneous nature of the comparison study. In addition to that, we measured constant shifting from sitting to standing position, which showed a protective element on arthritis. We did not find any studies assessing the combined effect of sitting and standing. However, literature shows that sitting for >2 hours daily protects against arthritis [28]. Therefore, there is a need to assess the combined effect of constant shifting from standing to sitting positions among the rural populations. Finally, more studies will be vital to measure the dose-response relationship on repetitive joint movements towards the development of arthritis.

The cross-sectional nature of this study might bring bias. However, in some instances we could seek medical records to avoid recall bias. Additionally, this study was conducted in one part of Nyamira County, and therefore, it cannot be generalized to other remote parts of Kenya with different economic and social activities. The strength of this study is that we explored a variety of risk factors, used a representative sample, and utilized a standardized data collection method.

## Conclusion

The prevalence of arthritis was found to be very high. Age, gender, employment status, area of residence, previous joint injury, hypertension, standing of > 2 hours per day, and constant shift from sit to stand positions were vital predictors of arthritis. Besides that, participants also had a high arthritis knowledge score. Therefore, there is a need for pragmatic, evidence-based strategies to prevent modifiable risk factors of arthritis.

### What is known about this topic

- *Arthritis is a significant public health problem affecting many people globally;*
- *There are many types of arthritis; however, the common ones are rheumatoid arthritis and osteoarthritis;*
- *Risk factors for arthritis are either modifiable or non-modifiable*.

### What this study adds

- *We found a high prevalence of arthritis among residents in rural areas of Nyamira County, Kenya;*
- *This study is among a few conducted in the remote areas of Kenya assessing the predictors of arthritis;*
- *Our Kaplan-Meier analysis curve showed that males had a shorter median age of developing arthritis compared to females*.
